# 18F-FDG-PET/CT in Initiation and Progression of Inflammation and Infection

**DOI:** 10.4274/mirt.18291

**Published:** 2017-06-01

**Authors:** Türkan Ertay, Mine Sencan Eren, Meral Karaman, Gülgün Oktay, Hatice Durak

**Affiliations:** 1 Dokuz Eylül University Faculty of Medicine, Department of Nuclear Medicine, İzmir, Turkey; 2 Dokuz Eylül University Faculty of Medicine, Department of Clinical Microbiology, İzmir, Turkey; 3 Dokuz Eylül University Faculty of Medicine, Department of Clinical Biochemistry, İzmir, Turkey

**Keywords:** abscess, Infection, inflammation, S. aureus, turpentine, positron emission tomography/computed tomography, 18F-2’-deoxy-2-fluoro-d-glucose, rat

## Abstract

**Objective:**

Detection/localization of infection and inflammation is important for the initiation of correct treatment as well as its maintenance. Nuclear medicine imaging methods play an important role in determining infection and inflammation. ^18^F-2’-deoxy-2-fluoro-d-glucose (^18^F-FDG) positron emission tomography/computed tomography (PET/CT) is highly sensitive in such cases when used with tomographic cross-sections. In this study, the development and progression of infection and inflammation were monitored on rats by using ^18^F-FDG via PET/CT.

**Methods:**

Sterile and infected abscesses were formed on rats using turpentine and *S. aureus*, respectively. For evaluation of the formation and progression of the abscess, ^18^F-FDG was injected into the rats and they were imaged by PET/CT at intervals of twenty-four hours for five days. Maximum standard uptake value (SUV_max_) of ^18^F-FDG was calculated.

**Results:**

The highest activity involvement was seen on the first day of abscess formation. On the first day, SUV_max_ of the *S. aureus* abscess was 3.9±0.9 while in the sterile abscess SUV_max_ in the first day was 2.2±0.8. ^18^F-FDG uptake decreased day by day and it reached the background level on the fourth and fifth days. There were statistically significant differences between *S. aureus* and sterile abscess, and between sterile abscess and background activity in terms of SUV_max_ values during the first three days (p<0.05). On the fourth and fifth days, there was no statistically significant difference between *S. aureus* and sterile abscess, and between sterile abscess and background activity (p>0.05).

**Conclusion:**

The results demonstrated that the SUV_max_ value for ^18^F-FDG can be useful in the early differentiation of sterile and infected abscess. In addition, ^18^F-FDG-PET imaging has the advantage of local availability of equipment and labeled agents leading rapid diagnosis of differentiation of infection and inflammation.

## Abstract (tr)

**Amaç:**

Enfeksiyon ve enflamasyonun tespit edilmesi ve yerinin belirlenmesi, doğru tedavinin başlanması ve hastaların bakımı için birincil öneme sahiptir. Enfeksiyon ve enflamasyonu saptamada nükleer tıp görüntüleme yöntemleri önemli rol oynar. ^18^F-2’-deoksi-2-fluoro-d-glukoz (^18^F-FDG) pozitron emisyon tomografisi/bilgisayarlı tomografi (PET/BT) yüksek duyarlılığa sahiptir. Bu çalışmada enfeksiyon ve enflamasyonun oluşum ve gelişim süreci ^18^F-FDG ile PET/BT kullanılarak izlendi.

**Yöntem:**

Terebentin ve *S. aureus* kullanılarak ratlarda steril ve enfekte apse oluşturuldu. Apsenin oluşum ve gelişim sürecini değerlendirmek için ratlar 24 saatlik aralar ile 5 gün ^18^F-FDG enjekte edilerek PET/BT ile görüntülendi. ^18^F-FDG’nin maksimum standart uptake değeri (SUV_maks_) hesaplandı.

**Bulgular:**

En yüksek aktivite tutulumu apse oluşumunun ilk gününde görüldü. İlk gün *S. aureus* için SUV_maks_ 3,9±0,9 iken steril apse için SUV_maks_ 2,2±0,8 idi. ^18^F-FDG tutulumu günden güne azalırken dördüncü ve beşinci günlerde zemin aktivite düzeyi kadar azaldı. *S. aureus* ile steril apse arasında ve steril apse ile background aktivite arasında SUV_maks_ değerleri açısından ilk üç gün görüntülerde istatistiksel olarak anlamlı fark vardı (p<0,05). Dördüncü ve beşinci günlerde ise *S. aureus* ile steril apse arasında ve steril apse ile kontrol arasında istatistiksel olarak anlamlı bir fark yoktu (p>0,05).

**Sonuç:**

Bu sonuçlar ^18^F-FDG SUV_maks_ değerlerinin erken dönemde steril ve enfekte apse arasındaki ayrım için yararlı olabileceğini öne sürmektedir. Ayrıca ekipmanların ve işaretli ajanların lokal olarak temin edilmesi enfeksiyon enflamasyon ayrımında ^18^F-FDG-PET görüntüleme yönteminin hızlı tanı yöntemi olmasını sağlar.

## INTRODUCTION

Detecting the presence and identifying the localization of infection and inflammation have primary importance for implementation of proper treatment and patient follow-up (1,2,3,4,5,6,7). Nuclear medicine offers powerful non-invasive imaging techniques for visualization of infection and inflammation-related disorders by imaging the whole body, thus enabling determination of both the localization and extent of inflammatory foci (8,9). Various methods have been developed that display different stages of the inflammatory response. Many radiopharmaceuticals have been evaluated extensively in both preclinical and clinical studies as potential diagnostic agents to identify the sites of infection (10,11,12,13). Although there are several imaging agents, only a few of them are being used in routine clinical practice. There is a definite role of ^18^F-2’-deoxy-2-fluoro-d-glucose (^18^F-FDG) in assessing disease extent, disease activity in patients with infection and inflammation, and evaluation of response to treatment (6,14). The high tissue radioactivity after administration of ^18^F-FDG corresponds to increased glucose uptake and consumption through the hexose monophosphate shunt, which is the main source of energy for chemotaxis and phagocytosis (7,15). ^18^F-FDG, an analog of glucose, is taken up by living cells via cell membrane glucose transporters and subsequently it is phosphorylated with hexokinase inside most cells. Activation of phagocytes, also known as respiratory burst activation, lead to increased ^18^F-FDG uptake (10). In sterile inflammation, administered ^18^F-FDG is mainly taken up by neutrophils and macrophages (6,15). A high degree of ^18^F-FDG uptake is detected in neutrophils during the acute phase of inflammation, while macrophages and polymorphonuclear leukocytes uptake ^18^F-FDG during the chronic phase (1,6,11,16). ^18^F-FDG is phagocytized by macrophages and phagocytic cells via d-glucose transporter. Through glycolysis, ^18^F-FDG is phosphorylated by hexokinase resulting in ^18^F-FDG-6 phosphate. Positron emission tomography (PET) imaging can be used alone or in conjunction with computed tomography (CT) in diagnosing and management of therapy planning in a variety of disorders (9).

The use of PET/CT represents the new generation in diagnostic modality. PET imaging detects an increase in metabolic activity while CT provides anatomic correlation (15,16,17,18). Inflammatory cells have an increased positive expression of glucose transporters and growth factors, which affect the affinity of these transporters for deoxyglucose. Due to structural analogy, 2-deoxyglucose ^18^F-FDG is uptaken at the site of infection at a high level depending on the rate of glycolysis. ^18^F-FDG is carried into the cells by the glucose transporters. In case of infection and inflammation, leukocyte activation occurs and glucose is used in the activation as an energy source. Glucose transporter receptors are stimulated by uptake of glucose and its analogues (17,19).

The aim of this study is to evaluate the development of infection and inflammation, as well as to monitor sterile and infected abscesses in rats by using ^18^F-FDG-PET/CT.

## MATERIALS AND METHODS

All animals were treated in accordance with the protocols approved by the Animal Care and Use Committee of the University. The designed study was conducted at the animal care facility of the Faculty of Medicine, Dokuz Eylül University.

In this study, sterile abscess was induced by using turpentine and infected abscess was induced by using *Staphylococcus aureus* ATCC 25923 strain on rats. Three groups of rats were used for imaging as sterile, infected, and control groups. Another group of rats were used to remove the abscess tissue to compare number of living organism with standard uptake value (SUV). They were male White Wistar Rats, clinically healthy animals of 150-220 gr body weight.

### Bacterial Strain and Rat Model for Abscess Formation

*S. aureus* strain (ATCC 25923) was grown in 5-10% sheep blood agar (Salubris, USA) after incubating overnight at 37°C.

One loop of the *S. aureus* colonies was suspended in Mueller Hinton broth (mhb) containing tube to obtain l07 colony-forming units (CFU)/mL.

The animals were anesthetized by intraperitoneal administration of xylazine, 5 mg/kg and ketamin, 35 mg/kg. For infected abscess formation on rats (n=14), *S. aureus* 0.5 mL 10^7^ CFU/mL was inoculated in the right arm of the rats subcutaneously. For sterile abscess formation on rats (n=7) 0.2-0.4 mL turpentine (Sigma-Aldrich) was injected into the right arm of the rats subcutaneously. In the control group (n=6), 0.5 mL 0.9% NaCl was injected into the right arm of the rats subcutaneously. Following each imaging, an abscess of a rat was removed in the another *S. aureus* group and living bacterial organisms were counted in the excised tissue.

### Positron Emission Tomography/Computed Tomography Imaging of Rats

A preliminary study was performed to optimize imaging time depending on abscess formation. First day image was acquired 24 hours after inoculation of *S. aureus* and turpentine. ^18^F-FDG (37 MBq) was injected intravenously via the tail vein. Prior to ^18^F-FDG injection, rats fasted for 4 hours and were well hydrated. Imaging was performed using PET/CT (PHILIPS Gemini TF), beginning one hour after injection of ^18^F-FDG on the first day for five days with an interval of twenty-four hours. ^18^F-FDG rat imaging was done with two-minute bed positions. On the first day, imaging was performed at the first and second hours after injection of ^18^F-FDG to obtain optimum imaging time. Non-diagnostic CT images were obtained (90 kVp and 30 mAs, with a thickness slice of 2 mm, the rotation time was 0.5 sec, 39 mm/sec bed speed, 512x512 matrix).

### Image Analysis

PET/CT images were visually and semi-quantitatively assessed. For semi-quantitative analysis of the PET images, a region of interest (ROI) was drawn around the abscess area on the right arm. In the control group, a ROI was drawn around a similar area as the background on the right arm. SUV_max_ was obtained from the images for evaluation of glucose metabolism of infection and inflammation detected by ^18^F-FDG-PET/CT.

The SUV_max_ of ^18^F-FDG uptake were calculated on abscess sites by using the formula:

SUV=Tissue concentration (Bq/g)/[injected dose (Bq)/body weight (g)]

## RESULTS

Twenty-four hours after inoculation of *S. aureus* or turpentine, swelling was apparent in the abscess site. A soft tissue infection developed on the right arm within twenty-four hours after bacterial inoculation. Swelling and redness of the abscess area were apparent in all rats. Abscess sites were visualized by ^18^F-FDG-PET/CT. A higher abscess/background ratio was detected at the first hour compared to the second hour after injection of ^18^F-FDG. Imaging time was chosen as the first post injection hour for the following days.

The initial SUV_max_ for *S. aureus* was 3.9±0.9 on the first day, while it was 2.2±0.8 for sterile abscess and 1.2±0.5 for control group rats. The first day SUV_max_ on the second hour following ^18^F-FDG injection was 2.8±0.6 for infected abscess, 1.9±0.9 for sterile abscess, and 1.2±0.09 for control group. During the following two days, although activity involvement decreased at *S. aureus* it was still higher than involvement in turpentine abscess. There were statistically significant differences between *S. aureus* and sterile abscess, and between sterile abscess and control group as SUV_max_ for the first three days (p<0.05). On the fourth and fifth days, there was no statistically significant difference between *S. aureus* and sterile abscess, and between sterile abscess and control group (p>0.05).

It was observed that SUV_max_ of infected abscess was higher than that of sterile abscess in all images during the first three days. CFUs per milliliters (CFU/mL) in excised abscess tissue was 10^4^ CFU/mL, 10^3^ CFU/mL, 10^2^ CFU/mL in the first, second and third days, respectively. There was no living organism at the fourth and fifth days. A correlation was detected between ^18^F-FDG activity and number of living microorganisms in excised abscess tissue.

**Figure 1:**
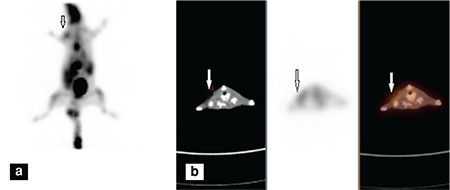
Sterile abscess imaging in rats by using ^18^F-2’-deoxy-2-fluoro-d-glucose positron emission tomography/computed tomography, (a) 3D MIP image, (b) Transverse cross-sectional images, computed tomography, positron emission tomography and fusion images

**Figure 2:**
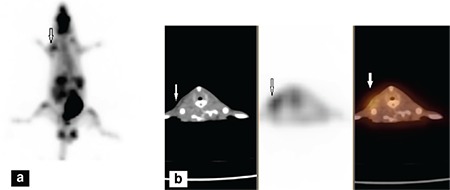
Infected abscess imaging in rats by using ^18^F-2’-deoxy-2-fluoro-d-glucose positron emission tomography/computed tomography, (a) 3D MIP image, (b) Transverse cross-sectional images, computed tomography, positron emission tomography and fusion images

Figures 1a, 1b, 2a, 2b display ^18^F-FDG images of sterile and infected abscess in rats.

Table 1 presents SUV_max_ for sterile abscess, infected abscess, and control rats as well as the quantity of living organisms. 

## DISCUSSION

It is clinically important to distinguish infection from inflammation (6,14). Abscess is a life threatening and important complication of inflammation or major surgery (19,17). Most infectious and inflammatory foci can be visualized accurately with radiolabeled autologous leukocytes. *In vitro* labeled leukocyte imaging is the gold standard for imaging most infection. However, preparation of this radiopharmaceutical is laborious, time consuming and requires handling of potentially contaminated blood (4). New agents are being developed that could potentially differentiate between infection and non-microbial inflammation. In addition to these, it is suggested that ^18^F-FDG-PET imaging can be used to visualize inflammatory foci when a high spatial resolution is required (1).

Kumar et al. (20) compared ^67^Ga Citrate SPECT and ^68^Ga Citrate PET in *S. aureus* infection in the rat model. They concluded that ^68^Ga Citrate PET is a faster imaging method as ^68^Ga has a half-life of 68 minutes compared to 78.3 hours for ^67^Ga. Yamada et al. (21) studied ^18^F-FDG uptake and its distribution in turpentine induced inflammatory tissue on male Donryu rats. They showed that the uptake in inflammatory tissue increased gradually upto 60 minutes and then decreased. Our study also showed an increased activity within 60 minutes followed by a decreased activity 120 minutes after injection of ^18^F-FDG. They reported an increasing activity which peaked on the 4 day after inoculation followed by a slow down. Our study detected the highest ^18^F-FDG activity a day after turpentine inoculation. In our study, there was also a correlation between ^18^F-FDG activity and quantity of living microorganisms in excised abscess tissue. These differences may be due to the rat’s immune tolerance.

Kaim et al. (22) studied ^18^F-FDG and ^18^F-FET in an acute phase abscess model. Their histological study showed increased ^18^F-FDG uptake that corresponded to cellular inflammatory infiltrates, mainly consisting of granulocytes. The necrotic abscess center and the second necrotic tissue layer were characterized by decreased ^18^F-FDG uptake. They documented a marked increase in ^18^F-FDG uptake at the site of infection, which could be attributed to activated granulocytes and macrophages. In their study, ^18^F-FET uptake was low in inflammatory infiltrates consisting of neutrophil, granulocytes and macrophages. They interpreted this finding as either low uptake in the bacterial area may be lacking bacterial uptake by ^18^F-FET or small number of living bacteria in the infection area. On the other hand, our study demonstrated that there was a correlation between bacterial load and ^18^F-FDG uptake rate. A greater bacterial load in the excised abscess yielded a higher SUV.

Sugawara et al. (23) compared ^18^F-FDG, thymidine, L-methionine, ^67^Ga Citrate and ^125^I-HSA in sites of bacterial infection in rats infected with *E. coli*. Their auto-radiographic study detected the highest ^18^F-FDG uptake in the inflammatory area of cell infiltration surrounding the necrotic region. In their study, ^18^F-FDG showed much higher uptake values than ^67^Ga Citrate or ^125^I-HSA. They reported lower methionine and thymidine accumulation in the infectious foci than ^18^F-FDG. In their bacterial model, an abscess was formed and the necrotic area showed slightly higher ^18^F-FDG uptake than the surrounding edematous muscle, while the center of the abscess in turpentine model showed very low ^18^F-FDG uptake. In our study, ^18^F-FDG uptake in the bacterial site was higher than the inflammation site. It has been reported that abscess-forming bacteria utilize glucose as an energy source using various pathways. The lack of any ^18^F-FDG uptake could be attributed to a low number of inflammatory cells, lack of granulation tissue, or absence of microorganisms.

Dumarey et al. (24) used ^18^F-FDG labeled leukocytes for imaging of inflammation. They found high sensitivity and specificity rates for the diagnosis of infection. They concluded that due to ^18^F-FDG uptake in the brain, the genitourinary tract and variable activity in the myocardium, bone marrow, stomach and bowel, ^18^F-FDG-PET had poorer diagnostic performance compared with labeled leukocytes for the detection of infection in these sites. We also observed major uptake in these organs. We agree with Dumarey et al. (24) that the diagnostic performance of ^18^F-FDG imaging in infection of these sites is poor. However, leukocyte labeling procedure is time consuming and needs careful handling process.

Pellegrino et al. (25) carried out a study to compare the relative uptakes of ^18^F-FDG and of ^18^F-FDG labeled WBCs in sterile and septic inflammation foci in an animal model. Their results showed that ^18^F-FDG-WBC PET imaging had a greater performance versus ^18^F-FDG in the sterile inflammation model as well as in E.coli and *P. aeruginosa* septic models. Jamar et al. (26) emphasized that it should be kept in mind that the choice between ^18^F-FDG imaging and an alternative technique depends on the need for rapid diagnosis and local availability of required equipment and labeled agents.

In our study, the highest ^18^F-FDG activity was observed on the first day following inoculation in the infection and the inflammation sites, and then the activity decreased day by day. This could be related to the rapid metabolic activity in fast recovering rats. On the fourth and fifth days, ^18^F-FDG activity was equal to that of the controls. We compared activity rates according to bacterial load by excising tissue. It was seen that bacterial load and ^18^F-FDG uptake values correlated with infection severity. In this study, the imaging was performed by PET/CT that is designed for human body imaging, if imaging studies had been obtained with small animal imaging device (microPET/CT) resolution of the images would have been higher than the presented images.

## CONCLUSION

Inflammatory and infected tissues utilize glucose as energy source for chemotaxis and phagocytosis. Therefore, the accumulation of ^18^F-FDG as a glucose analog can be used for diagnosis of infection and inflammation. Sterile and infected abscess differentiation can be evaluated by imaging with ^18^F-FDG-PET at early phase. The value of SUV_max_ explores correlation between sterile abscess and infected abscess. ^18^F-FDG-PET is also a useful technique to understand the extent of the infection and inflammation process. In addition to PET imaging method with ^18^F-FDG has the possibility of rapid diagnosis and easier with the advantage of local availability.

## Figures and Tables

**Table 1:**
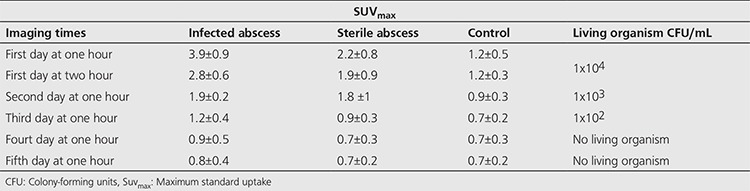
Maximum standard uptake values for sterile abscess, infected abscess and control and number of living organism
